# Dietary L-Methionine modulates the gut microbiota and improves the expression of tight junctions in an in vitro model of the chicken gastrointestinal tract

**DOI:** 10.1186/s42523-024-00303-w

**Published:** 2024-03-19

**Authors:** Min-Jin Kwak, Anna Kang, JuYoung Eor, Sangdon Ryu, Youbin Choi, Jung Min Heo, Minho Song, Jong Nam Kim, Hyeon-Jin Kim, Younghoon Kim

**Affiliations:** 1 Department of Agricultural Biotechnology and Research Institute of Agriculture and Life Science, Seoul National University, Seoul, 08826 Korea; 2 Divisions of Environmental Materials, Honam National Institute of Biological Resources (HNIBR), 58762, Mokpo, Korea; 3 Department of Food Science & Nutrition, Dongseo University, Busan, 47011 Korea; 4 CJ Cheiljedang, 330, Dongho-ro, Jung-gu, Seoul, 04560 Korea

**Keywords:** FIMM, *C. elegans*, Methionine, Microbiome, Poultry

## Abstract

**Background:**

The poultry industry encounters a number of factors that affect growth performance and productivity; nutrition is essential for sustaining physiological status and protecting against stressors such as heat, density, and disease. The addition of vitamins, minerals, and amino acids to the diet can help restore productivity and support the body’s defense mechanisms against stress. Methionine (Met) is indispensable for poultry’s energy metabolism, physiology, performance, and feed utilization capacity. Through this study, we aimed to examine the physiological effects of methionine supplementation on poultry as well as alterations of intestinal microbiome.

**Methods:**

We utilized the DL- and L- form of methionine on *Caenorhabditis elegans* and the FIMM (Fermentor for intestine microbiota model) in-vitro digesting system. A genomic-analysis of the transcriptome confirmed that methionine supplementation can modulate growth-related physiological metabolic pathways and immune responses in the host poultry. The *C. elegans* model was used to assess the general health benefits of a methionine supplement for the host.

**Results:**

Regardless of the type or concentration of methionine, supplementation with methionine significantly increased the lifespan of *C. elegans*. Feed grade L-Methionine 95%, exhibited the highest lifespan performance in *C. elegans*. Methionine supplementation increased the expression of tight junction genes in the primary intestinal cells of both broiler and laying hens, which is directly related to immunity. Feed grade L-Methionine 95% performed similarly or even better than DL-Methionine or L-Methionine treatments with upper doses in terms of enhancing intestinal integrity. In vitro microbial cultures of healthy broilers and laying hens fed methionine revealed changes in intestinal microflora, including increased *Clostridium*, *Bacteroides*, and *Oscillospira* compositions. When laying hens were given feed grade L-Methionine 95% and 100%, pathogenic *Campylobacter* at the genus level was decreased, while commensal bacteria were increased.

**Conclusions:**

Supplementation of feed grade L-Methionine, particularly L-Methionine 95%, was more beneficial to the host poultry than supplementing other source of methionine for maintaining intestinal integrity and healthy microbiome.

## Background

In the poultry industry, various factors including genetics, environment, and nutrition could affect broiler chickens’ growth performance and productivity [1–3]. In particular, nutrition has a vital role for maintaining the physiological barrier from diverse stress factors, such as heat, density, and diseases [4]. These stressors can negatively affect production performance by incrementing the nutritional demand via the activation of systemic immune responses and defense systems [5, 6]. Accordingly, dietary supplementation of vitamins, functional minerals, and amino acids could be an effective approach to restoring productivity and supporting the defense process against various stressors in the poultry industry [7–9].

Methionine (Met), which is the first limiting amino acid in chickens, is crucial for protein deposition, energy metabolism, DNA expression and the antioxidant capacity in poultry [10–12]. Accordingly, dietary supplementation of Met in laying hens’ feed could improve reproduction efficiency and egg quality in laying hens, and it could also improve immune status by reducing oxidation stress [13, 14]. Moreover, various studies demonstrated that Met addition could increase the weight of breast and leg muscles by minimizing abdominal fat accumulation in broiler chicken [15].

Generally, there are two isomers (L- and D-form) of amino acids, and in order to be utilized in the animal’s body, D-isomer amino acids must be converted to the L-form mainly in the liver and kidney [16]. In the poultry industry, the L-form of Met (L-Met) and the combination of D-form and L-form of Met (DL-Met) have been mostly employed as a Met source in chicken feeds, and the ratio of the L-form and D-from has determined its bioavailability and functionality to regulate physiology and metabolism in the animal’s body [17–19]. D- and L-form methionine are utilized indiscriminately as animal feed in the domain. Still, Esteve-Garcia and Khan claim that there is not enough study being done at this time to determine the different effects caused by the D/L ratio of methionine; further studies will be needed to elucidate the conversion efficiency of D- to L-Met process [18]. Moreover, because the effects of L-Met and DL-Met on the intestinal environment are yet to be established, we aimed to determine the optimal composition of L-and D-form of methionine for poultry industry and to investigate the exact mechanisms of various types of Met on chicken’s gut microenvironment using diverse in vitro experimental techniques including *C. elegans* bio-replacement model, in vitro digesta fermentation model, and primary cell lines of broiler chicken and laying hens.

## Results

### Transcriptome analysis

According to the hepatic RNA-seq data archived from the SRA database of methionine-restrict feeding trials with mice and chickens, methionine restriction could affect various physiological metabolic pathways (Table 1). In particular, dietary methionine regulation could modulate energy-related metabolism, including insulin signaling pathway, bile secretion, AMPK signaling pathway, and carbon metabolism, and it also showed regulating effects on amino acid-related metabolism, such as biosynthesis of amino acid, glutathione metabolism, and glycine, serine, and threonine metabolism. Moreover, the transcriptome analysis result demonstrated that dietary methionine restriction could affect systemic immune responses by regulating T-cell activation (Table 2).

**Table 1:** Metabolic pathways of *gallus gallus* and *mus musculus* fed with methionine-restricted diets

Pathway	Total	Expected	Hits	P. Value	FDR
Metabolism of xenobiotics by cytochrome P450	66	0.287	6	3.35E-07	0.000105
Chemical carcinogenesis	94	0.409	6	2.75E-06	0.000432
Drug metabolism– cytochrome P450	68	0.296	5	1.01E-05	0.00106
Biosynthesis of amino acids	78	0.34	5	1.98E-05	0.00156
Drug metabolism– other enzymes	87	0.379	5	3.37E-05	0.00212
Metabolic pathways	1490	6.49	17	5.91E-05	0.00309
**Glutathione metabolism**	64	0.279	4	0.00016	0.00719
Platinum drug resistance	77	0.335	4	0.000328	0.0129
Glycine, serine and threonine metabolism	40	0.174	3	0.000672	0.0234
**Insulin signaling pathway**	40	2.09	10	0.00238	0.00062
Fluid shear stress and atherosclerosis	143	0.623	4	0.0033	0.104
Bile secretion	72	0.313	3	0.00369	0.105
**AMPK signaling pathway**	29	1.51	8	0.00398	0.00138
Folate biosynthesis	26	0.113	2	0.00561	0.147
Carbon metabolism	120	0.522	3	0.015	0.363
Riboflavin metabolism	8	0.0348	1	0.0343	0.77
Vitamin B6 metabolism	9	0.0392	1	0.0385	0.807

**Table 2:** Immunological pathways of *gallus gallus* and *mus musculus* fed with methionine-restricted diets

Pathway	Total	Expected	Hits	P. Value	FDR
**Regulation of T cell activation**	3	0.00796	1	0.00794	1
Proteolysis	5	0.0133	1	0.0132	1
One_carbon metabolic process	6	0.0159	1	0.0158	1
Phospholipid metabolic process	7	0.0186	1	0.0184	1
Negative regulation of sequence_specific DNA binding transcription factor activity	10	0.0265	1	0.0262	1
Cellular response to stress	12	0.0318	1	0.0314	1
Phosphatidylinositol biosynthetic process	13	0.0345	1	0.034	1
Regulation of cytokine production	16	0.0424	1	0.0417	1
Protein polyubiquitination	20	0.0531	1	0.0518	1
Peripheral nervous system development	20	0.0531	1	0.0518	1

### C. elegans lifespan analysis

**Fig. 1:**
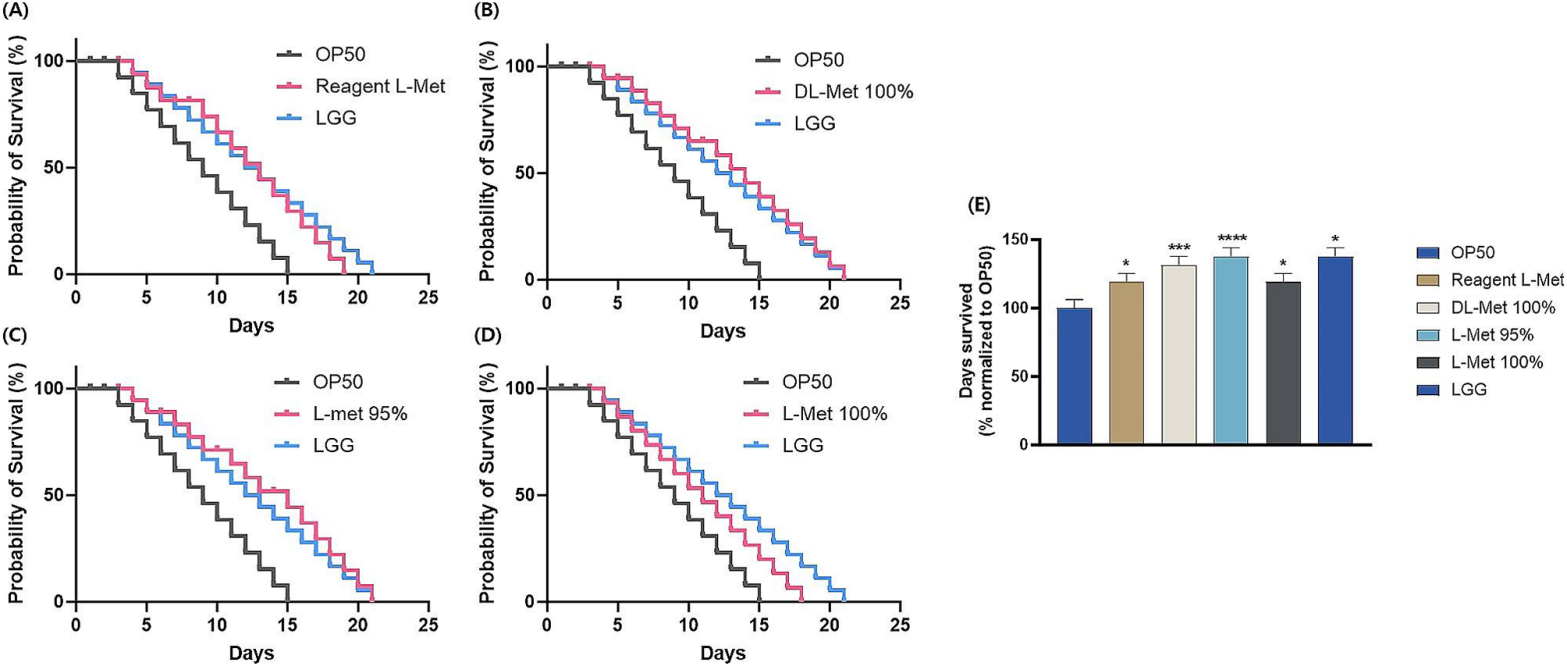
Methionine supplementation enhanced *C. elegans* longevity. (**A**-**D**) The lifespan assay of *C. elegans* supplemented with each methionine is evaluated and (**E**) the survival rate. All values are expressed as mean ± SD; significant differences were determined using one-way ANOVA followed by Student’s t-test at *P < 0.05, ***P < 0.001, ****P < 0.0001 compared with OP50

To investigate the biological function of various types of methionine, the lifespan assay using *C. elegans* model was performed. The results of this assay suggested that feeding of reagent grade L-Met and feed grade L-Met 100 showed increased longevity by 20% compared to *Escherichia coli* OP50 fed group. And the feed grade L-Met 95% and feed grade DL-Met 100% groups significantly increase the worms’ life span by 40% compared to OP50 fed group and their lifetime was as long as that of *Lacticaseibacillus rhamnosus* GG fed group. Collectively, dietary methionine supplementation, regardless of the source used, increased the lifespan of *C. elegans.* (Fig. 1).

### In vitro digestion analysis using FIMM model

**Fig. 2:**
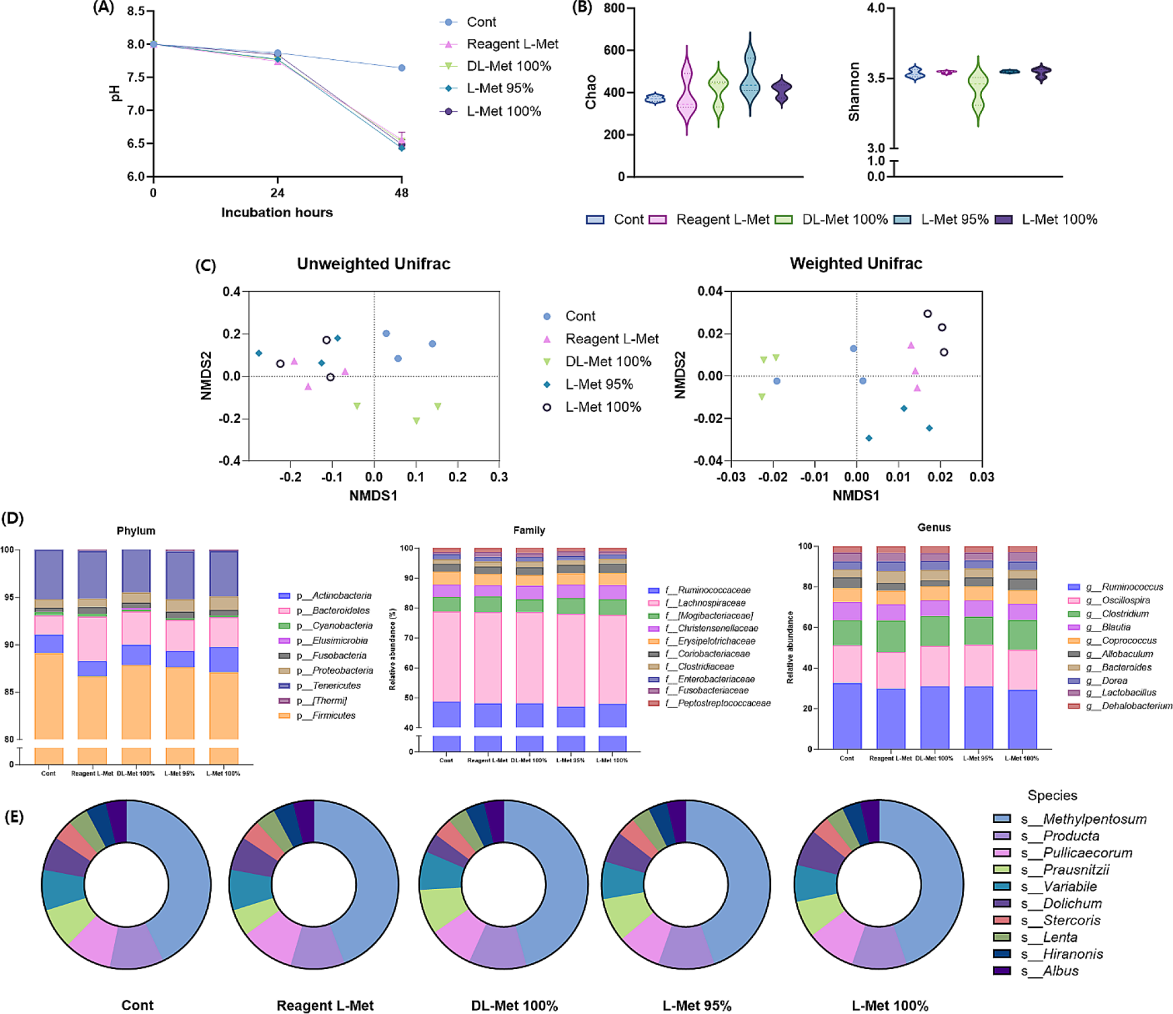
Methionine supplementation-induced microbial changes of broiler digesta. To confirm the practical application of methionine, the small intestine digest of healthy broilers was co-cultured with methionine using FIMM (in vitro microbial culture) and the intestinal microflora was analyzed. (**A**) The digesta pH changes were measured during incubation. (**B**) The richness of the digesta microbiota was assessed through alpha-diversity metrics, specifically employing Chao and Shannon indices. The results are presented as the mean ± SD. (**C**) To compare the diversity of the digesta microbiota across different groups, PCoA plots were utilized, grounded in both weighted and unweighted UniFrac distances pertaining to the microbiome of broilers digesta. Each plot symbolizes an individual replicate, with the axes depicting the two principal dimensions that capture the most significant variance within the microbial communities. (**D**) Compositional changes were analyzed under phylum, family, genus, and species level

We employed the novel FIMM model to analyze the modulating effect of various types of methionine on the gut microbiome of broiler chickens and laying hens. The results of the FIMM assay using intestinal digesta samples of broiler chickens showed significant reduced digesta pH (Fig. 2A) by various types of methionine treatment, and the β-diversity results showed that each methionine treatment stimulated its own unique microbiome population. However, they did not affect the α-diversity of microbial population of broiler chicken’s digesta samples (Fig. 2B, C). Moreover, the FIMM-digested microbial population showed a significantly increased population of *Bacteroidetes* at the phylum level, with the increment of the population of *Clostridium*, *Bacteroides*, and *Oscillospira* at the genus level (Fig. 2D).

**Fig. 3:**
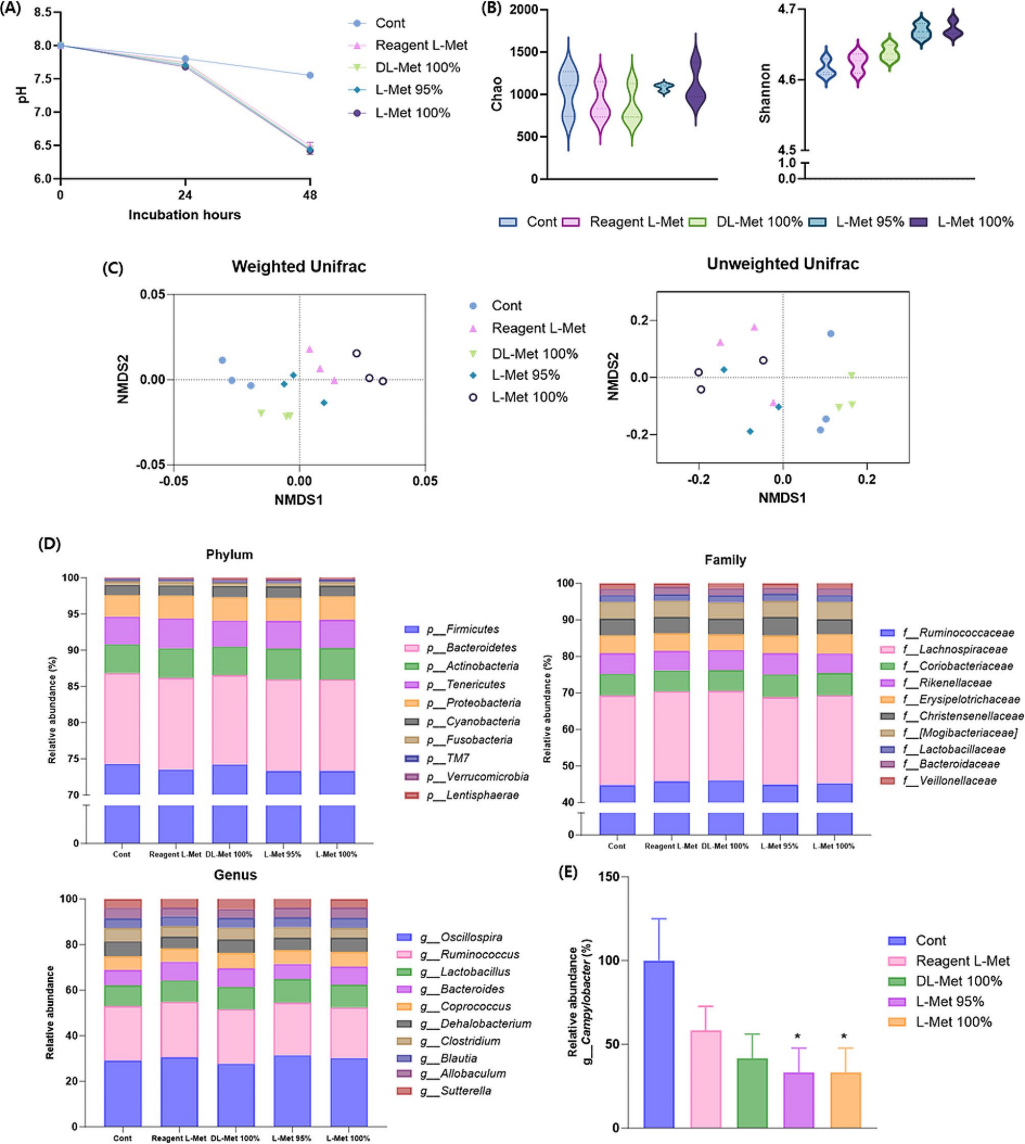
Methionine supplementation-induced gut microbiota changes of laying hen digesta. To confirm the practical application of methionine, the small intestine digest of healthy broilers was co-cultured with methionine using FIMM (in vitro microbial culture) and the gut microbiota was analyzed. (**A**) The digesta pH changes were measured during incubation. (**B**) The richness of the digesta microbiota was assessed through alpha-diversity metrics, specifically employing Chao and Shannon indices. The results are presented as the mean ± SD. (**C**) To compare the diversity of the digesta microbiota across different groups, PCoA plots were utilized, grounded in both weighted and unweighted UniFrac distances pertaining to the microbiome of laying hens digesta. Each plot symbolizes an individual replicate, with the axes depicting the two principal dimensions that capture the most significant variance within the microbial communities. (**D**) Compositional changes were analyzed under phylum, family, genus, and species level and (**E**) pathogenic population of *Campylobacter*. The values are expressed as mean ± SD; significant differences were determined using one-way ANOVA followed by Student’s t-test at *P < 0.05 compared with Cont

On the other hand, we also investigated the gut microbial modulation effects of methionine substrates in the FIMM digestion system using intestinal samples of laying hens. In vitro digestion of digesta samples of laying hens with various types of methionine also significantly decreased pH compared to that of the control group (Fig. 3A). The microbial diversity analysis revealed that digestion with feed grade L-Met 95% and 100% showed a significantly increased richness index of α-diversity compared to the control group, and the microbial population of each treatment had been diversified by co-digestion with diverse sources of methionine (Fig. 3B, C). At genus level, all sources of methionine addition increased the probiotic population in gut microbiome including *Oscillospira, Lactobacillus*, and *Corprococcus* compared to the control group. Furthermore, co-digestion of laying hens’ digesta showed a significantly decreased population of *Campylobacter* at the species level, and it is known as representative pathogenic bacteria in chicken as identified in various poultry diseases including spotty liver disease, necrotic hepatitis, egg production losses, and flock mortality (Fig. 3D, E).

### MTT assay with intestinal primary cells of broiler chickens and laying hens

To examine the toxicity and cell proliferation effects of methionine, the cell viability assay was carried out with intestinal epithelial primary cells of broiler chicken and laying hen. The results of this study demonstrated that the addition of 2 and 5 mM of feed grade L-Met 95% and 100% significantly increased the cell viability in broiler chicken’s primary cell and all sources of methionine accelerated cell proliferation by concentration-dependent manner in primary cells of laying hen.

**Fig. 4:**
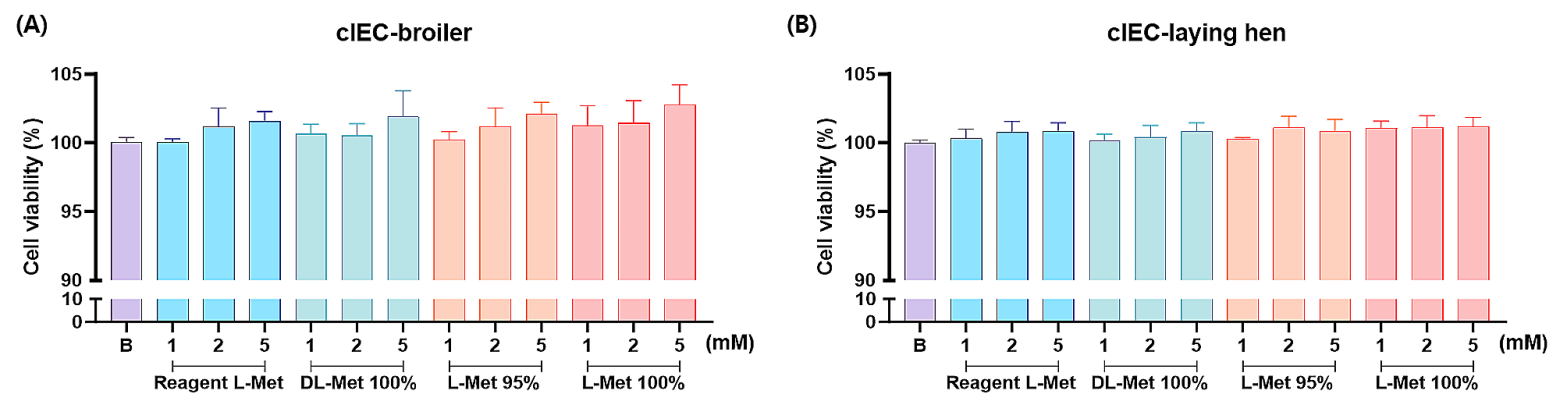
Cell viability assay of chicken primary intestinal epithelial cells to methionine treatment. MTT assay was performed to confirm the protective effect of methionine against glucose deficiency stress using primary epithelial cells isolated from intestinal tissues of (**A**) broiler (cIEC-broiler) and (**B**) laying hens (cIEC-laying hen). All values are expressed as mean ± SD. There was no significance between the datasets

Afterward, we also performed an additional cell viability assay to validate the protective effects of methionine on glucose deficiency stress using intestinal epithelial primary cells of broiler chickens and laying hens. In both primary cell lines, all sources of methionine supplementation restored the stress from glucose-deficiency, and feed grade L-Met 95% and 100% showed a cell proliferation accelerating effect in broiler chicken’s intestinal primary cells (Fig. 4).

### Gut gene expression profiles related to tight junction and immune responses

**Fig. 5:**
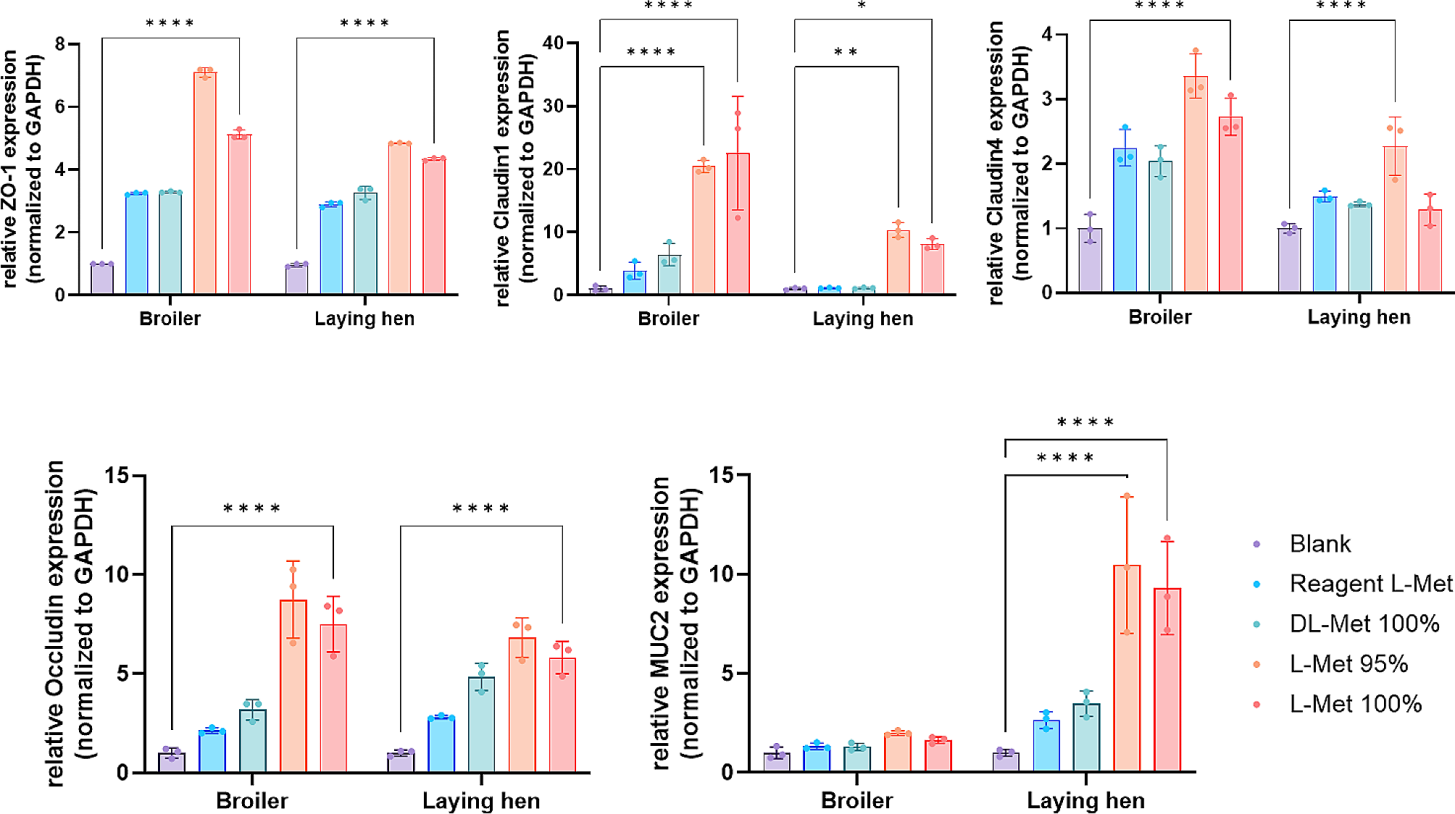
Relative expression of intestinal tight junction genes of methionine supplemented poultry primary intestinal epithelial cells. The gene expression levels related to intestinal tight junction were quantified using qRT-PCR and ∆C(t) values were normalized to internal control GAPDH. All values are expressed as mean ± SD; significant differences were determined using one-way ANOVA followed by Student’s t-test at *P < 0.05, **P < 0.005, ****P < 0.0001 compared to blank

**Fig. 6:**
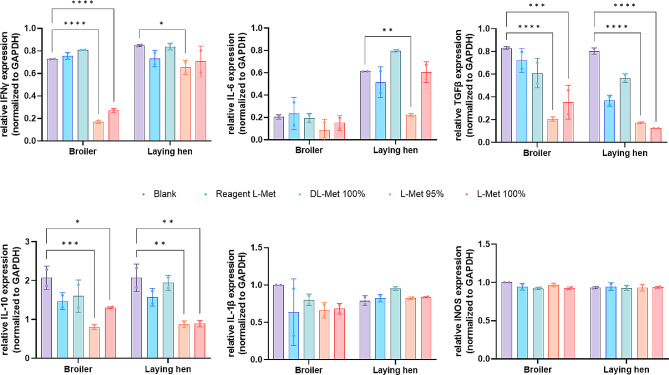
Relative expression of inflammatory cytokines presenting genes of methionine supplemented poultry primary intestinal epithelial cells. The gene expression levels related to pro- and anti-inflammatory cytokines were quantified using qRT-PCR and ∆C(t) values were normalized to internal control GAPDH. All values are expressed as mean ± SD; significant differences were determined using one-way ANOVA followed by Student’s t-test at *P < 0.05, **P < 0.005, ***P < 0.001, ****P < 0.0001 compared to blank

To elucidate the protective effects of methionine on intestinal primary cells, the gene expression levels related to tight junction proteins (*ZO-1, Claudin 1, Claudin 4, Occludin*, and *MUC2*) and inflammatory cytokines (*IFN-γ, IL-6, TGF-β*, and *IL-10*) were examined. In both primary cell lines of broiler chicken and laying hens, the addition of feed grade L-Met 95% and 100% significantly increased the gene expression levels of *ZO-1* and *Claudin 1* compared to the control group, and particularly the feed grade L-Met 95% showed a significantly higher expression level of *Claudin 4* and *Occludin* compared to the control group in both primary cell lines. Moreover, the mRNA level of *MUC2* was significantly increased by feed grade L-Met 95% and 100% supplementations only in laying hens (Fig. 5). On the other hand, supplementation of feed grade L-Met 95% and 100% showed a significant decrement in the expression level of the anti-inflammatory cytokine, *IL-10*, and pro-inflammatory cytokine, *TGF-β*, compared to the control group. Additionally, feed grade L-Met 95% treatment significantly decreased the expression level of *IFN-γ* and *IL-6* in laying hens, and the level of *IFN-γ* in broiler chicken was also decreased by feed grade L-Met 95% and 100% treatments (Fig. 6).

## Discussion

Methionine is an essential amino acid that has been widely utilized in the animal realm as a supplement for better growth performance [19]. It is necessary for life and must be ingested since animals cannot synthesize methionine by themselves in order to produce the necessary quantity of proteins. Alongside cysteine, methionine can assist animals maintain a healthy level of fat and body weight by controlling fat metabolism. It can also encourage growth and renewal of body tissue by improving the animal’s capacity to utilize nutrients [12, 20].

Through a genomic analysis of the transcriptome, we confirmed that methionine supplementation can modulate the physiological metabolic pathways and immune responses that are directly related to the growth performance of the host poultry. Due to the fact that methionine supplementation is required for animal feed, we endeavored to determine the variety of methionine that provides the greatest host-health and economic benefits. *C. elegans* was used in this study to evaluate the general health benefits of a methionine supplement to the host. *E. coli* OP50, which is widely believed to have no effect on the metabolism of *C. elegans*, and LGG, which are widely regarded as the most effective probiotics in the world, were used as control groups to determine the effect of each form of methionine. Compared to other animal models, *C. elegans* has a relatively limited lifespan, with a reproductive cycle that lasts only a few days. Therefore, *C. elegans* has been usefully utilized as an efficient animal replacement model that can rapidly demonstrate health improvement effects [21, 22]. Supplements of methionine significantly increased the lifespan of *C. elegans*, regardless of the source or percentage of methionine; feed grade L-Met 95%, which is composed of a single type of methionine, the L-type, with 5% of Corynebacterium fermentation medium from the production process, demonstrated the best performance of *C. elegans* lifespan [22, 23]. It is yet to be determined that what component from the Corynebacterium fermentation medium promoted the lifespan of *C. elegans*.

In accordance with the beneficial effects of L-methionine on poultry gut microbiota, our investigation extended to its impact on primary intestinal cells of poultry, effectively reflecting the genuine state of the poultry’s internal environment. Given that our study employs primary intestinal cells, our attention centered on the markers of tight junctions, serving as proxies for intestinal integrity [24, 25]. All sources of methionine supplementation increased tight junction gene expression levels in primary intestinal cells from both broiler and laying hens, which is directly associated with immunity because increased tight junction is associated with low pathogen invasion and growth performance by absorption of sufficient nutrients [6, 26, 27]. When compared to the feed grade DL_Met or high amounts of methionine supplement groups in both broiler and laying hens, feed grade L-Met performed similarly or even better in terms of improving intestinal integrity and immune response.

To evaluate the impact of methionine supplementation on poultry gut microbiota under real-world conditions, the FIMM (in vitro microbial culture) technique was employed to co-culture digesta from the entire small intestines and ceca of healthy broilers and laying hens with various sources of methionine. The small intestine and cecum of chickens fulfill distinct but complementary functions in the digestive process and nutrient assimilation. Specifically, the small intestine is the primary site for the digestion and absorption of nutrients, supported by a microbiota that facilitates these processes. Conversely, the cecum functions as a fermentation site, breaking down complex carbohydrates and hosting a varied and abundant microbiota essential for immune regulation and gut health [28–30]. Given the critical roles both the small intestine and cecum play in the nutritional efficiency and health of chickens, comprehensive analysis via FIMM was conducted on the digesta from these two digestive compartments of each bird.

A notable decline in the pH of the culture medium was observed during the FIMM procedure, underscoring the importance of maintaining appropriate pH levels for the sustenance of bacterial cultures. Extremes in pH, either too high or too low, are detrimental to bacterial growth [31]. In this study, the pH of the culture medium was employed as a benchmark for gauging the bacterial uptake efficiency of methionine. Typically, methionine stabilizes the pH within a range of 5.5 to 6.1, suggestive of a neutral pH [32]. Therefore, a pH threshold of 6.5 was established as indicative of effective bacterial utilization of methionine and was selected as the endpoint for this investigation. This particular pH level is deemed favorable for bacterial viability and is likely to maintain oral cavity neutrality upon consumption by animals, suggesting a benign impact on the intestinal microflora [33, 34].

Following the observed pH reduction, alterations in the fecal microbiota of both broilers and laying hens were noted under various methionine treatment conditions. Although there was no significant change in alpha diversity, all sources of methionine treatments increased the compositions of *Bacteroides*, *Oscillospira*, and *Clostridium* in broilers, underscoring the pivotal roles these commensal bacteria play within the chicken gastrointestinal tract [35–37]. These bacteria contribute extensively to the host’s health, nutrient absorption, and disease resistance mechanisms. Specifically, *Bacteroides* species are instrumental in breaking down complex carbohydrates into short-chain fatty acids (SCFAs), which are essential for energy provision, maintaining gut pH balance, and inhibiting the growth of pathogenic microbes [38–40]. *Oscillospira*, associated with healthy weight and metabolic outcomes, is believed to aid in the fermentation process and the production of beneficial metabolites, although the precise mechanisms remain to be fully elucidated [41, 42]. *Clostridium*, known for both its beneficial and pathogenic strains, includes species that are crucial for fermentation, SCFA production, and the synthesis of essential vitamins like vitamin B12, critical for the host’s metabolic functions [43–46]. The increase in these commensal bacteria from methionine treatments highlights their significance in enhancing nutrient utilization, gut health, and immune system modulation, emphasizing the need for further exploration of these microbial interactions to improve poultry health management and production efficiency.

The meticulous administration of methionine, notably in the form of feed-grade L-Met at concentrations of 95% and 100%, has been demonstrated to significantly mitigate the prevalence of *Campylobacter* species within the gastrointestinal tract of laying hens. These species are notorious pathogens implicated in causing substantial foodborne illnesses in humans, despite their asymptomatic manifestation in poultry hosts [37, 47, 48]. Concurrently, this intervention fosters the proliferation of advantageous microbial communities, exhibiting a composition distinctively varied from those observed in broilers, prominently including genera such as *Oscioospira*, *Lactobacillus*, and *Coprococcus* [37, 49, 50]. Among these, *Coprococcus* is particularly noteworthy for its commensal attributes, contributing to the enhancement of gut health through its role in fermentation processes and the synthesis of SCFAs [51, 52]. This dynamic underscores the imperative for nuanced strategies aimed at harmonizing microbial populations within the poultry gut, thereby augmenting poultry health and safeguarding food safety through a comprehensive understanding of microbial ecology.

## Conclusions

Overall, we studied the intestinal integrity-immunity enhancing and microbiota diversifying effect of methionine supplementation utilizing *C. elegans* and poultry samples from this study. This study demonstrates that supplementation of L-methionine showed beneficial effect on intestinal integrity, immunity, and microbiome composition, albeit the mechanisms behind the positive effects are yet to be elucidated.

## Materials and methods

### Genomic analysis for methionine-related host response

Transcriptome datasets were obtained from previous studies from Gene expression omnibus (GEO) database of National Center for Biotechnology Information (NCBI) and GEO RNA-seq experiments interactive navigator (GREIN). RNA-seq data were selected from the GEO database. The datasets GSE165993, GSE181220, and GSE98039 were meticulously chosen for their relevance to species *Gallus gallus* and *Mus musculus*, and their insights into the effects of dietary methionine restriction. GSE165993 and GSE181220 contribute valuable data on systemic metabolic pathways and the immune-regulatory effects of dietary methionine through the analysis of 24 mouse colon tumor and 4 small intestine biosamples [53]. Meanwhile, GSE98039 provides a detailed transcriptome profile of 20 biosamples of chicken liver and pectoralis major muscles under methionine-restricted diets [54], offering a comprehensive view of methionine’s role in metabolic and tissue responses. Sample comparatives were analyzed for differentially expressed genes (DEG) using NetworkAnalyst 3.0 web-server (https://www.networkanalyst.ca/NetworkAnalyst/home.xhtml) as well as limma workflow, which is designed for gene expression experiment data. Functional gene set enrichment analysis (GSEA) was performed to classify common biological processes and metabolic pathways for DEGs annotation. Gene ontology (GO); Kyoto encyclopedia of genes and genomes (KEGG); and Reactome pathway enrichment analysis of DEGs were analyzed by employing NetworkAnalyst. Gene expression differences between chickens and mice were standardized using the limma package (version 3.46.0), applied to TMM-normalized read counts [55, 56]. To address the issue of multiple comparisons, p-values underwent adjustment via False Discovery Rate (FDR) correction, classifying genes as differentially expressed when the FDR was below 0.05. Furthermore, a gene was identified as having a moderate effect size if its Log Fold Change (LFC) exceeded 1, indicating a significant alteration in expression levels between the two species.

### C. elegans lifespan assay and methionine preparation

The *C. elegans* strain *fer-15;ferm-1* mutant was used, and they were routinely maintained on Nematode Growth Medium (NGM) agar plates. Eggs were extracted in sodium hypochlorite–sodium hydroxide solution and synchronized L1 worms were grown on NGM agar plates seeded with *Escherichia coli* OP50 at 25 °C to obtain sterile L4/young adult worms [57]. To perform a lifespan assay with series of methionine samples, the L1 or L4 stage of *C. elegans* strain *fer-15;fem-1* were individually transferred with a platinum wire onto 35-mm-diameter NGM agar plates. The control group was administered *E. coli* OP50 (hereafter OP50; approximately 8.0 × 10^9^ culture-forming units/mL (CFU/mL)), and the experimental group was exposed to OP50 with various types of methionine. For each lifespan assay, 90 worms per treatment were assayed on three plates (30 worms per plate) and incubated at 25 °C. The number of live *C. elegans* was counted daily and transferred to a new plate every day, and the assay was conducted until all *C. elegans* died. Reagent grade L-Methionine 100% (L-Met) was obtained from Sigma-Aldrich (MO, USA), and feed grade DL-Met (D-met 50%, L-Met 50%), feed grade L-Met 95% (L-Met Eco, CJ Cheiljedang, Seoul, Republic of Korea), and feed grade L-Met 100were donated by CJ % (CJ Cheiljedang, Seoul, Republic of Korea).

### Poultry primary intestinal cell isolation

In this study, primary intestinal cells were isolated from broilers (*n* = 4, aged 10 weeks) and laying hens (*n* = 4, aged 25 weeks) provided by the Daejeon Poultry Research Unit of Chungnam National University (Daejeon, South Korea). Following euthanasia, the entire small intestine, including the ceca, was harvested. The small intestine tissues were decomposed immediately for the isolation of primary intestinal epithelial cells, and the digesta from the entire small intestine and ceca were frozen at -80℃ for the subsequent assay.

To isolate the intestinal epithelial cells of poultry, the intestine tissues were cleansed of fat and mucus in ice-cold Hank’s balanced salt solution (HBSS, Gibco; NY, USA) medium. Then they were digested in digestion medium for 30 min at 37℃ and the digested tissues get centrifuged at 100 g for 3 min. The particle was resuspended in washing medium at 37 °C and then filtered through a Falcon (AZ, USA) 100 m and 40 m cell-strainer. The recovered aggregates were resuspended in basal medium and plated on 24-well matrigel-coated plates (Corning; MA, USA) [58].

### Cell viability and quantitative reverse transcription PCR

To determine the potential toxicity of methionine treatments on the intestinal epithelial cells of poultry, a cell viability assay was conducted under two conditions: DMEM/F12 high glucose medium (Gibco) and glucose starvation stress [59, 60]. For the glucose starvation stress, the intestinal epithelial cells of poultry were incubated in DMEM/F12 without glucose supplement for 24 h and then supplemented with each methionine for another 24 h. Then, the cells were harvested to ascertain the gene expression levels associated with intestinal tight junction and inflammatory cytokines [61]. According to the manufacturer’s instructions, total RNA was extracted with TRIZOL reagent (Invitrogen; CA, USA) and purified with RNeasy Mini Kit (Qiagen; Hilden, Germany). The CFX96 Real-Time equipment (Bio-rad; CA, USA) and miScript SYBR Green PCR kits were used to practice qRT-PCR.

### Fermentor for intestine microbiota model (FIMM)

The FIMM system simulates a intestine-like environment; during incubation, digest-like media with the proper pH, temperature, and anaerobicity restore the bacteria flora in the sample, which was damaged during sampling [62]. For the FIMM assay, strain-specific pools of the entire intestinal and ceca digesta were aseptically homogenized in filter bags (3 M, Saint Paul, MN, USA) using a stomacher (JumboMix, Interscience, Saint Nom, France). The supernatant was collected and inoculated 10% to the FIMM medium in triplicate, while each methionine supplement except the control was added to a final concentration of 5 mM and incubated for 48 h. The FIMM medium was prepared using modified Gifu anaerobic medium (mGAM, HIMEDIA, DB Maarn, Netherlands), which has been regarded as the standard medium for anaerobic bacteria [63]. The medium pH was altered to 8.0 and the temperature was set to 42 °C to resemble the conditions in the poultry intestine [64, 65].

### Metagenomic analysis using 16 S rRNA sequencing

Following the FIMM assay, samples from each culture were collected in triplicate for metagenomic analysis. The DNeasy PowerSoil Pro Kit (Qiagen, Hildesheim, Germany) was utilized to extract gDNA. The V4 region of the 16 S rRNA genes was amplified (V4 amplicon primer sequences: forward, 5’-TCGTCGGCAGCGTCAGATGTGTATAAGAGACAGGTGCCAGCMGCCGCGGTAA; reverse, 5’-GTCTCGTGGGCTCGGAGATGTGTATAAGAGACAGGGACTACHVGGGTWTCTAAT) through V4 amplicon PCR. Each PCR product was indexed using Nextera XT Index Kit (Illumina, Inc; SD, USA) and sequenced using Illumina iSeq 100 (Illumina, Inc) [66].

Samples exhibiting Phred quality scores lower than Q30 were omitted from subsequent analyses. The Fastq files, derived from iSeq paired-end sequencing, were processed using Mothur (version 1.48.0) following the standard operating procedures outlined in the Mothur user guide. This involved an initial step of error mitigation through the exclusion of misaligned sequences via comparison with the SILVA v138.1 database, which aids in the consolidation of infrequent sequences differing by one or two base pairs into more substantial sequences [67, 68]. Chimeric sequences were identified and eliminated using the vsearch tool. Taxonomic categorization was conducted utilizing databases formatted for Greengenes, updated in 2013, to discard sequences not classified under archaea and bacteria. Singleton sequences were removed through the “split.abund” subroutine in Mothur, and operational taxonomic units (OTUs) were defined at a 0.03 distance threshold, corresponding to 97% sequence similarity [68, 69].

For the analysis of microbial diversity, Mothur was utilized, employing OTU-based methods. Bacterial alpha diversity metrics, such as the Shannon and Chao indices, were assessed using a nonparametric one-way analysis of variance (ANOVA), specifically the Kruskal–Wallis test, via the “rarefaction” command [68, 70]. This was followed by Tukey’s post hoc analysis in instances where significant differences were detected (*p* < 0.05). To calculate phylogenetic diversity for UniFrac distance estimation, the “dist.seq” command was executed, and the resulting distance matrices were visualized with PCoA plots [71, 72]. Additionally, variations in the relative abundance of bacterial taxa were examined using Welch’s t-test, employing Prism 9 (GraphPad Software, CA, USA).

### Statistical analysis

All data points used in this study were collected in triplicate and expressed as the mean standard deviation based on three repeated experiments; significant differences were determined using the Student’s t-test and one-way ANOVA followed by the Turkey’s post hoc test. The *C. elegans* assay data were analyzed using the Kaplan-Meier method and visualized using Prism 9 (GraphPad Software, USA).

## Data Availability

The metagenomic datasets for digesta from broilers and laying hens supplemented with methionine can be accessed through the NCBI BioProject, identified by the accession number PRJNA1078227 and PRJNA1078238.

## Abbreviations

ANOVA: analysis of variance
CFU: culture-forming units
DEG: differentially expressed genes
DL-Met: D- and L-forms of methionine
FIMM: fermentor for intestine microbiota model
GEO: gene expression omnibus
HBSS: Hank’s balanced salt solution
L-Met: L-form of methionine
Met: methionine
mGAM: modified Gifu anaerobic medium
NGM: nematode growth medium
